# Correction: Casili et al. Therapeutic Potential of BAY-117082, a Selective NLRP3 Inflammasome Inhibitor, on Metastatic Evolution in Human Oral Squamous Cell Carcinoma (OSCC). *Cancers* 2023, *15*, 2796

**DOI:** 10.3390/cancers16061211

**Published:** 2024-03-20

**Authors:** Giovanna Casili, Sarah Adriana Scuderi, Marika Lanza, Alessia Filippone, Deborah Mannino, Raffaella Giuffrida, Cristina Colarossi, Marzia Mare, Anna Paola Capra, Federica De Gaetano, Marco Portelli, Angela Militi, Salvatore Cuzzocrea, Irene Paterniti, Emanuela Esposito

**Affiliations:** 1 Department of Chemical, Biological, Pharmaceutical and Environmental Sciences, University of Messina, Viale Ferdinando Stagno D’Alcontres, 31, 98166 Messina, Italy; gcasili@unime.it (G.C.); sarahadriana.scuderi@unime.it (S.A.S.); mlanza@unime.it (M.L.); afilippone@unime.it (A.F.); deborah.mannino@unime.it (D.M.); annapaola.capra@unime.it (A.P.C.); fedegaetano@unime.it (F.D.G.); angela.militi@unime.it (A.M.); salvator@unime.it (S.C.); eesposito@unime.it (E.E.); 2 IOM Ricerca, Via Penninazzo 11, 95029 Viagrande Catania, Italy; raffaella.giuffrida@grupposamed.com (R.G.); cristina.colarossi@grupposamed.com (C.C.); marzia.mare@grupposamed.com (M.M.); 3 Department of Biomedical and Dental Science, Morphological and Functional Images, University of Messina, Via Consolare Valeria, 98125 Messina, Italy; marco.portelli@unime.it

In the original publication [1], there was a mistake in Figure 4L as published. Figure 4I and Figure 4L were the same image, displayed with slightly different brightnesses. The corrected Figure 4 appears below.

The authors state that the scientific conclusions are unaffected. This correction was approved by the Academic Editor. The original publication has also been updated.

## Figures and Tables

**Figure 4:**
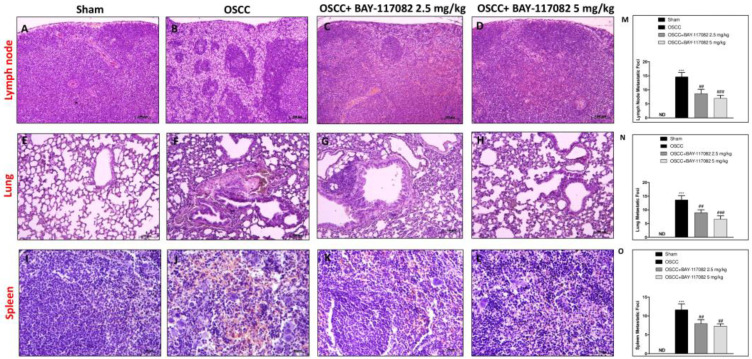
BAY-117082 treatment modulated the metastasis process in the lymph node, lung, and spleen. Histological analysis revealed that the treatment with BAY-117082 at doses of 2.5 and 5 mg/kg was able to reduce the degree of metastasis in the lymph node (**C**,**D**,**M**), lung (**G**,**H**,**N**), and spleen (**K**,**L**,**O**) in the C group compared with the OSCC group (**B**,**F**,**J**). (**A**,**E**,**I**) Control groups in the lymph node, lung, and spleen. Data are representative of at least three independent experiments. ND, not designed. *** *p* < 0.001 vs. sham; ### *p* < 0.001 and vs. ## *p* < 0.01 OSCC.
