# Supplementary material for: Therapeutic Potential of BAY-117082, a Selective NLRP3 Inflammasome Inhibitor, on Metastatic Evolution in Human Oral Squamous Cell Carcinoma (OSCC)
Source: Cancers (Basel). 2023 May 17;15(10):2796. doi: 10.3390/cancers15102796 (PMC10216277; doi:10.3390/cancers15102796)
Supplement: Supplementary file 1 [file cancers-15-02796-s001.zip › cancers-2378100-SI.pdf]

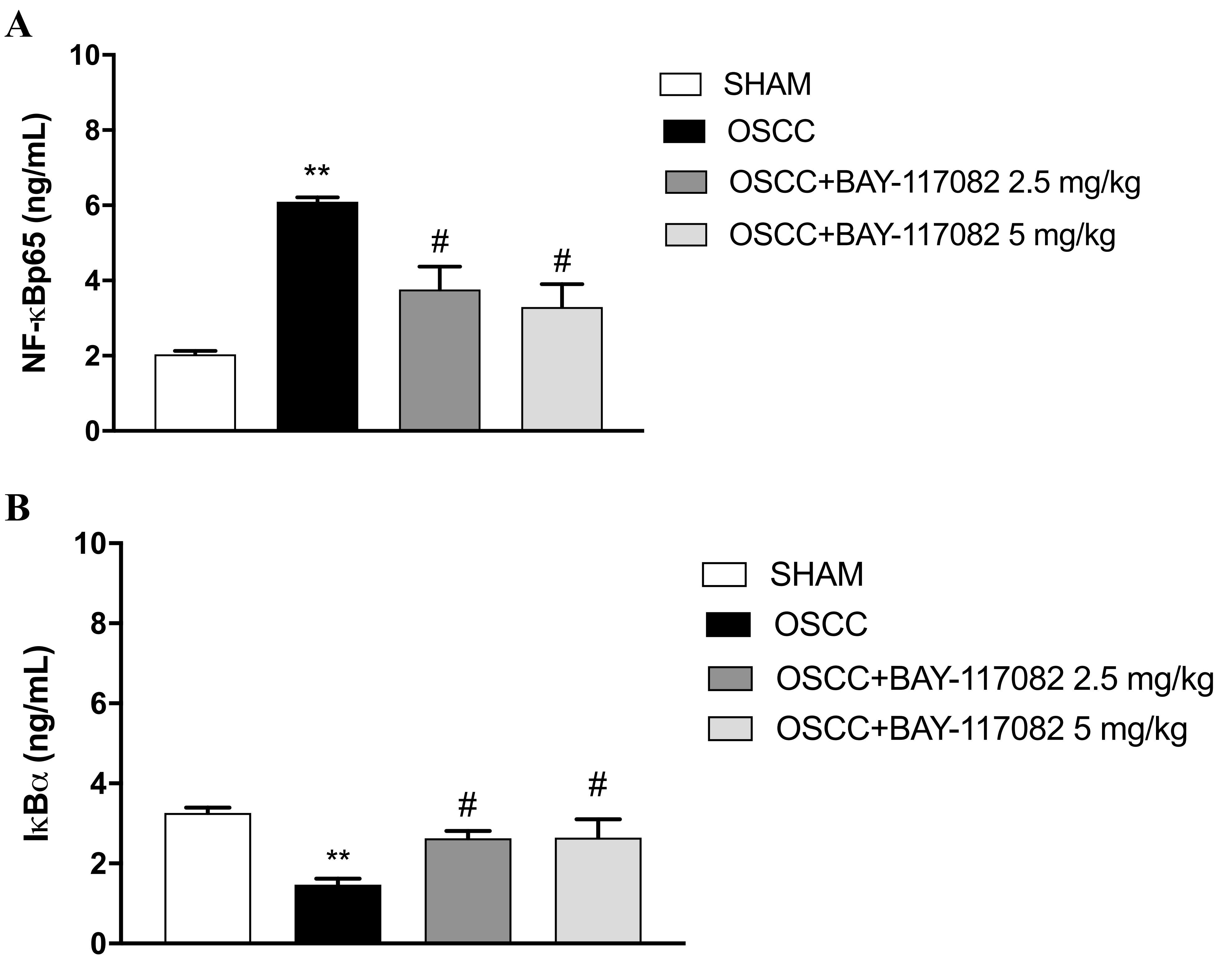

Supplementary Figure S1. Effect of BAY-117082 on NF-κB/IκBα pathway. ELISA kits revealed that BAY-117082 is able to significantly reduce NF-κB/IκBα pathway activation. (A) **p <0.01 vs Sham; #p < 0.05 vs OSCC; (B) **p <0.01 vs Sham; #p < 0.05 vs OSCC.

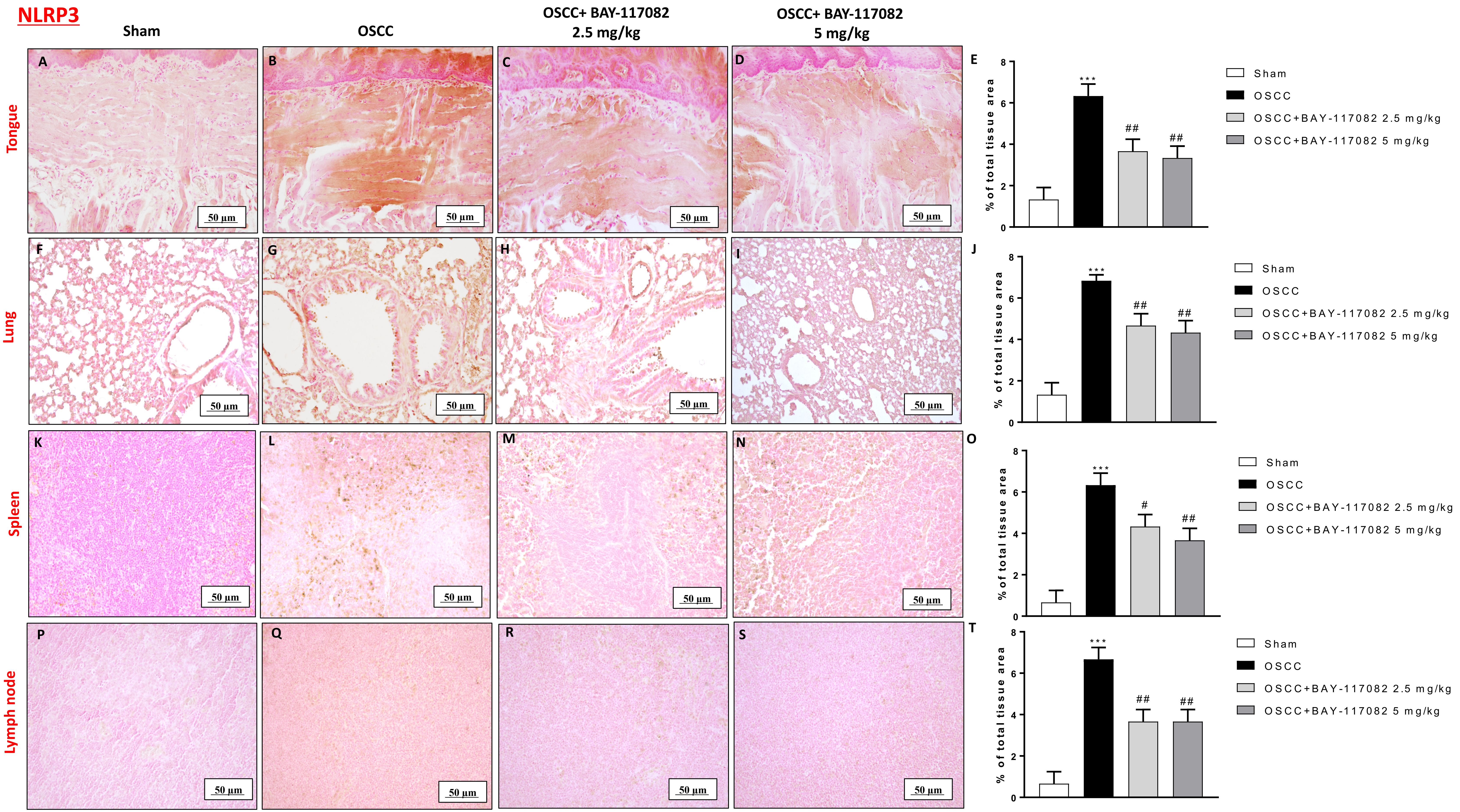

Supplementary Fig. S2. Effect of BAY-117082 on NLRP3 inflammasome. Immunoistochemical analysis demonstrated that BAY-117082 reduced cell-positive NLRP3 staining in tongue (C, D, E), lung (H, I, J), spleen (M, N, O) and lymph node tissue (R, S, T) compared to OSCC groups (B, G, L, Q). (E) ***p <0.001 vs Sham; ##p < 0.01 vs. OSCC. (J) ***p <0.001 vs Sham; ##p < 0.01 vs. OSCC (O) ***p <0.001 vs Sham; #p < 0.05 vs. OSCC; ##p < 0.01 vs. OSCC; (T) ***p <0.001 vs Sham; ##p < 0.01 vs. OSCC

Supplementary Figure S2

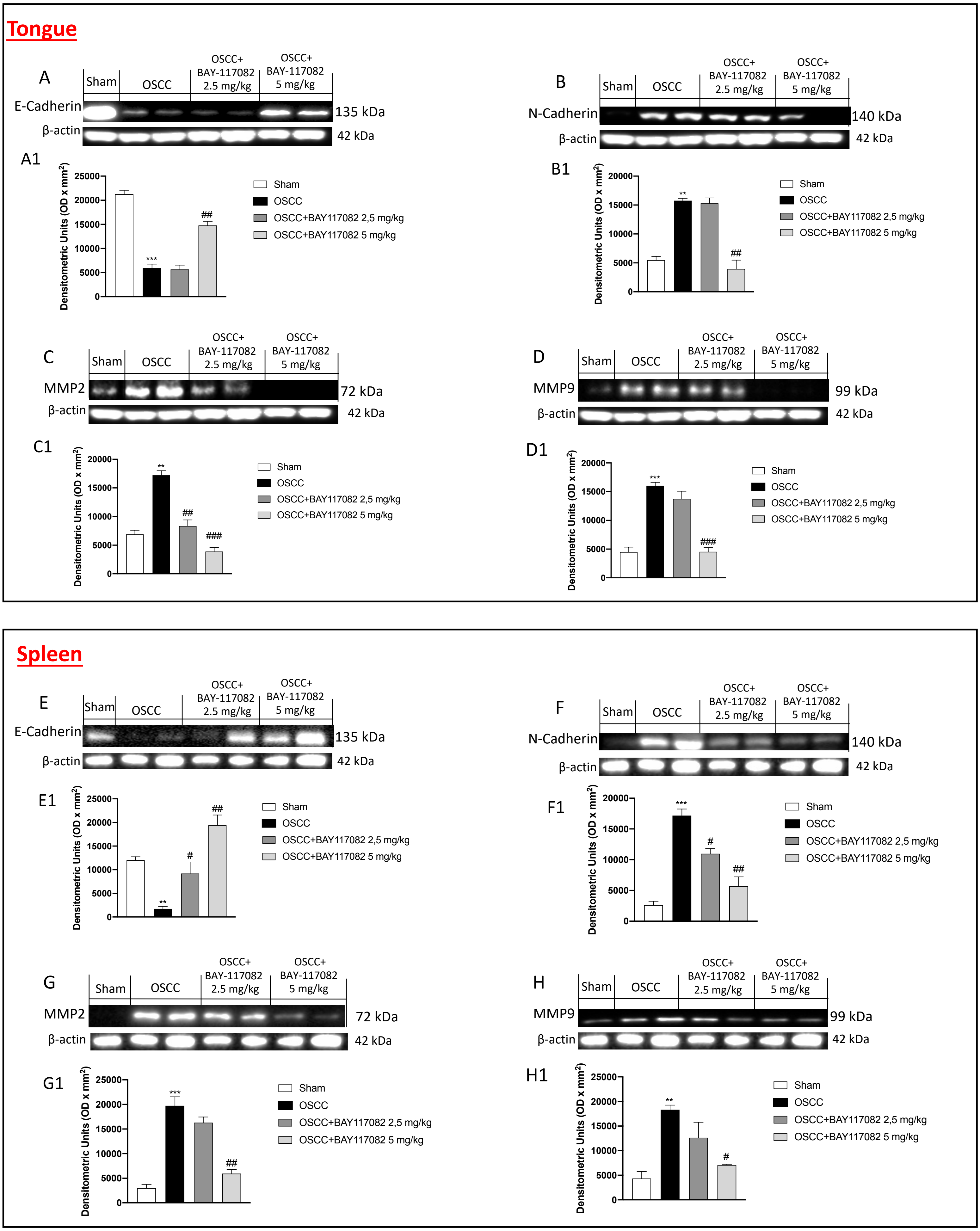

Supplementary Figure S3. Effect of BAY-117082 on E-cadherin, N-cadherin, MMP2 and MMP9 expression in tongue and spleen samples. The blots reveal that treatment with BAY-117082 was able to restore E-cadherin and reduce N-cadherin, MMP2 and MMP9 expression compared to OSCC group, in tongue (A, B, C and D) as well as in spleen (E, F, G and H). Data are representative of at least three independent experiments. (A, G) ***p <0.001 vs Sham; ##p < 0.01 vs. OSCC; (B) **p <0.01 vs Sham; ##p < 0.01 vs OSCC; (C) **p <0.01 vs Sham; ##p < 0.01 vs. OSCC; ###p < 0.001 vs. OSCC; (D) ***p <0.001 vs Sham; ###p < 0.001 vs. OSCC; (E) **p <0.01 vs Sham; #p < 0.05 vs OSCC; ##p < 0.01 vs OSCC; (F) ***p <0.001 vs Sham; #p < 0.05 vs OSCC; ##p < 0.01 vs. OSCC; (H) **p <0.01 vs Sham; #p < 0.05 vs. OSCC.

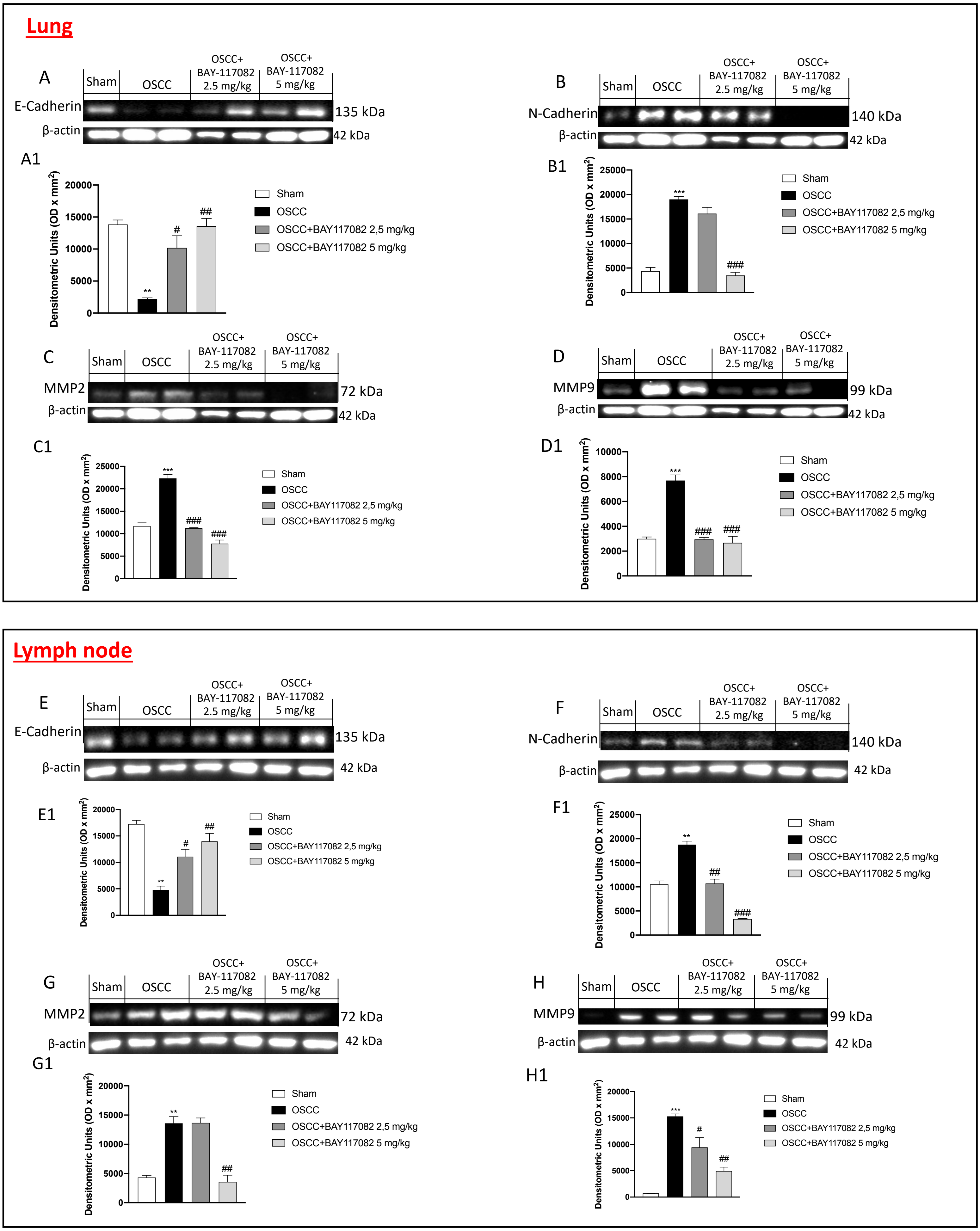

Supplementary Figure S4. Effect of BAY-117082 on E-cadherin, N-cadherin, MMP2 and MMP9 expression in lung and lymph node samples. The blots reveal that treatment with BAY-117082 was able to restore E-cadherin and reduce N-cadherin, MMP2 and MMP9 expression compared to OSCC group, in lung (A, B, C and D) as well as in lymph node (E, F, G and H) . Data are representative of at least three independent experiments. (A, G) **p <0.01 vs Sham; #p < 0.05 vs. OSCC; ##p < 0.01 vs. OSCC; (B, C, D) ***p <0.001 vs Sham; ###p < 0.001 vs. OSCC; (E) **p <0.01 vs Sham; #p < 0.05 vs. OSCC; ##p < 0.01 vs. OSCC; ##p < 0.01 vs OSCC;(F) **p <0.01 vs Sham; ##p < 0.01 vs. OSCC; ###p < 0.001 vs. OSCC; (H) ***p <0.001 vs Sham; #p < 0.05 vs. OSCC; ##p < 0.01 vs. OSCC.

Supplementary Figure S4

Original Western Blots
Tongue samples

NLRP3

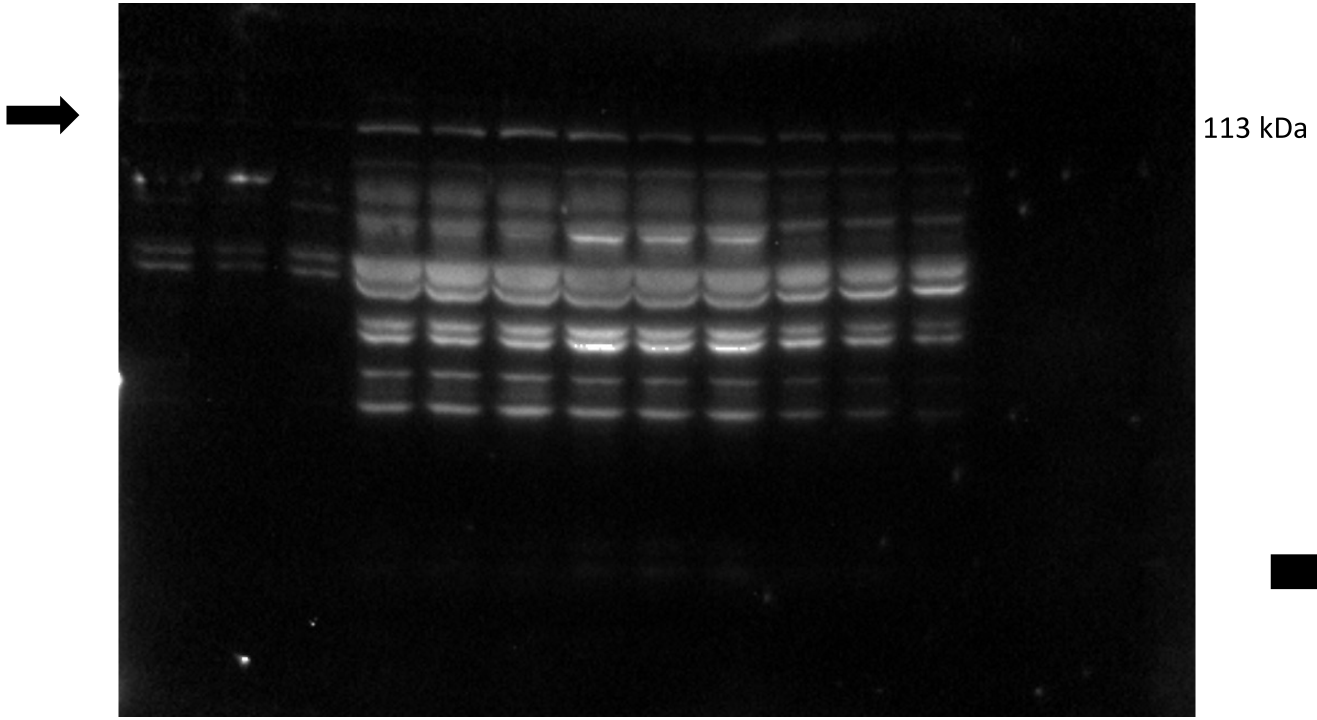

ASC

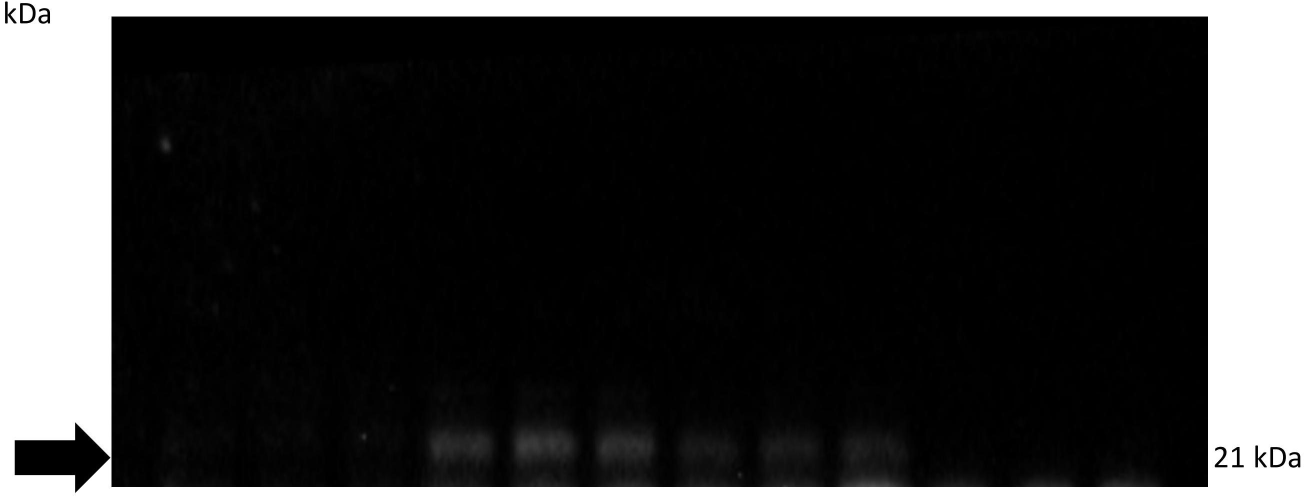

Original Western Blots
Tongue samples

IL-18

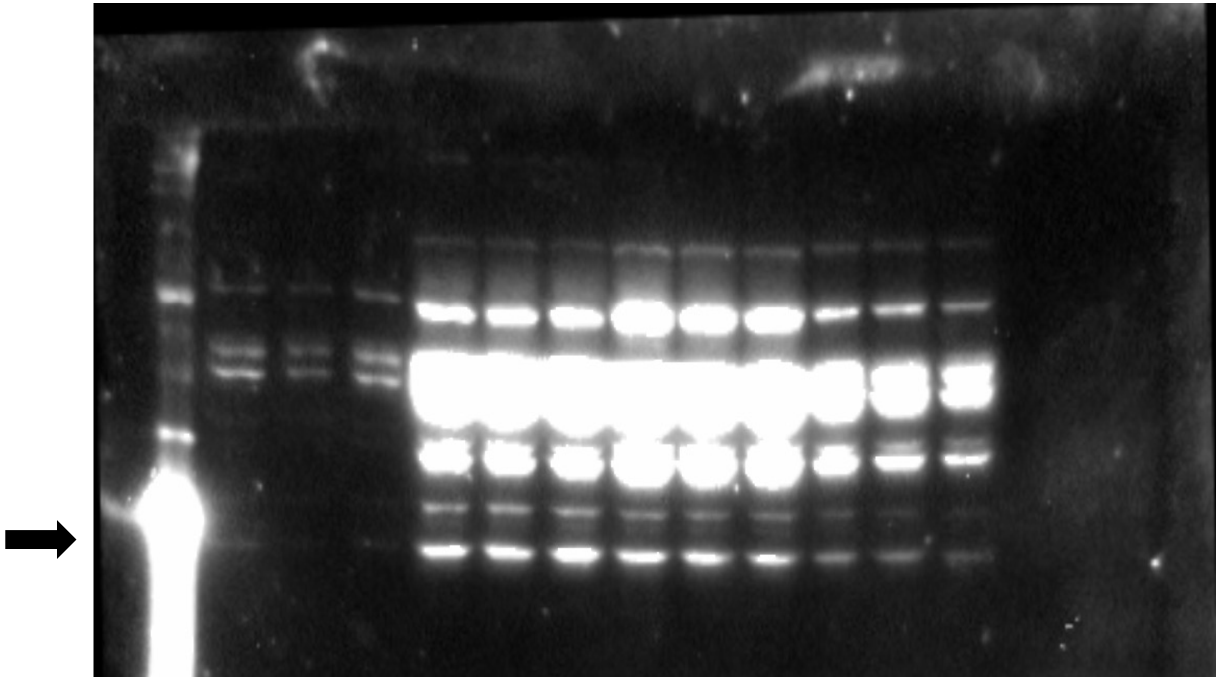

18 kDa

IL-1β

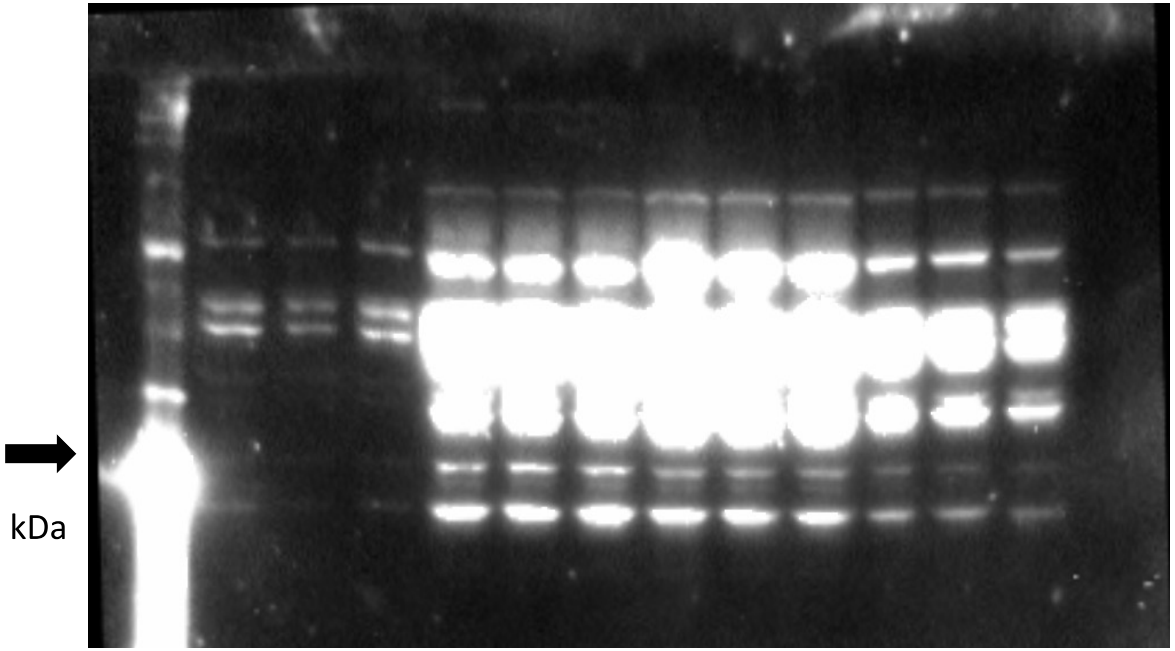

31 kDa

Original Western Blots
Tongue samples

β -actin for NLRP3, ASC, IL-1β and IL-18

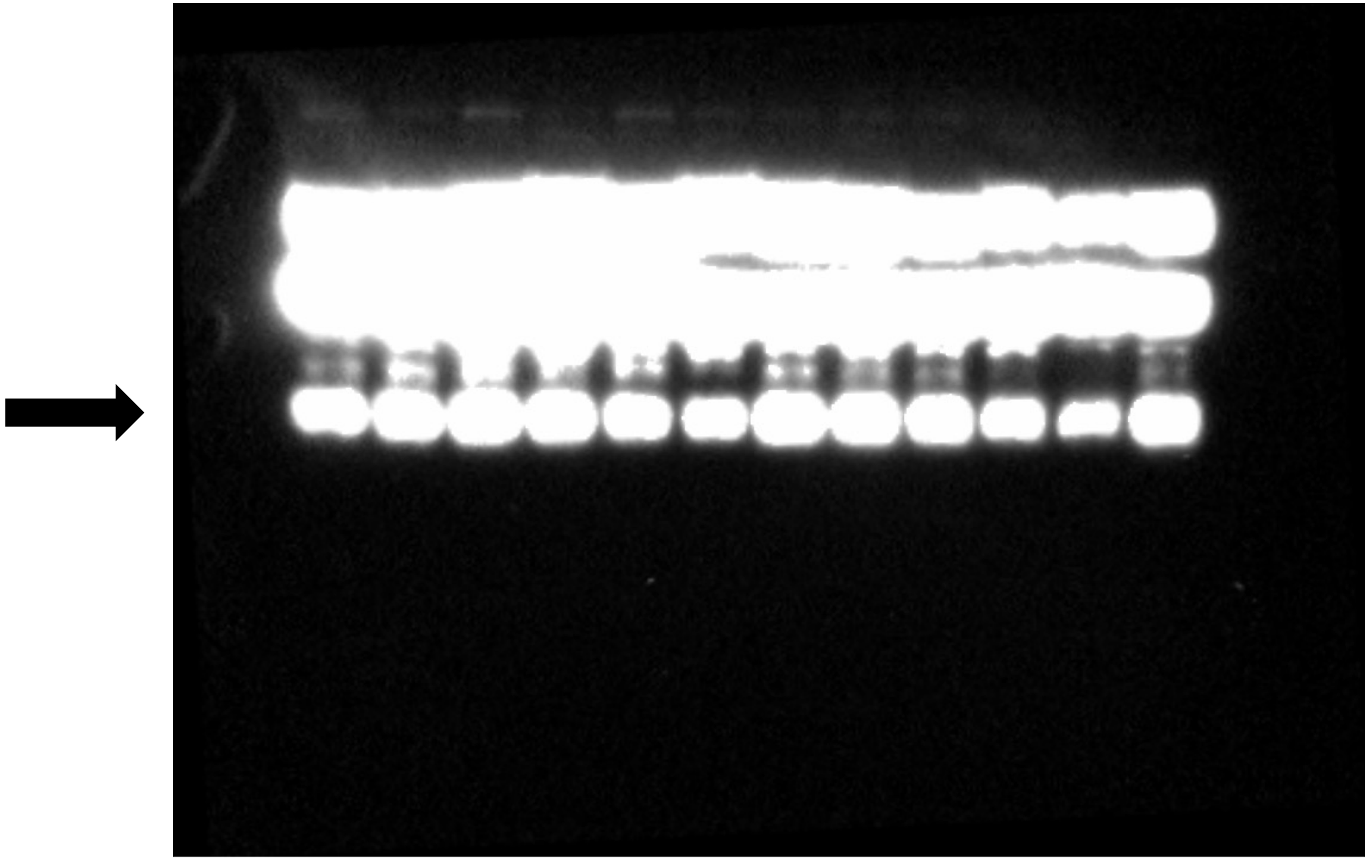

Original Western Blots
Lung samples

NLRP3

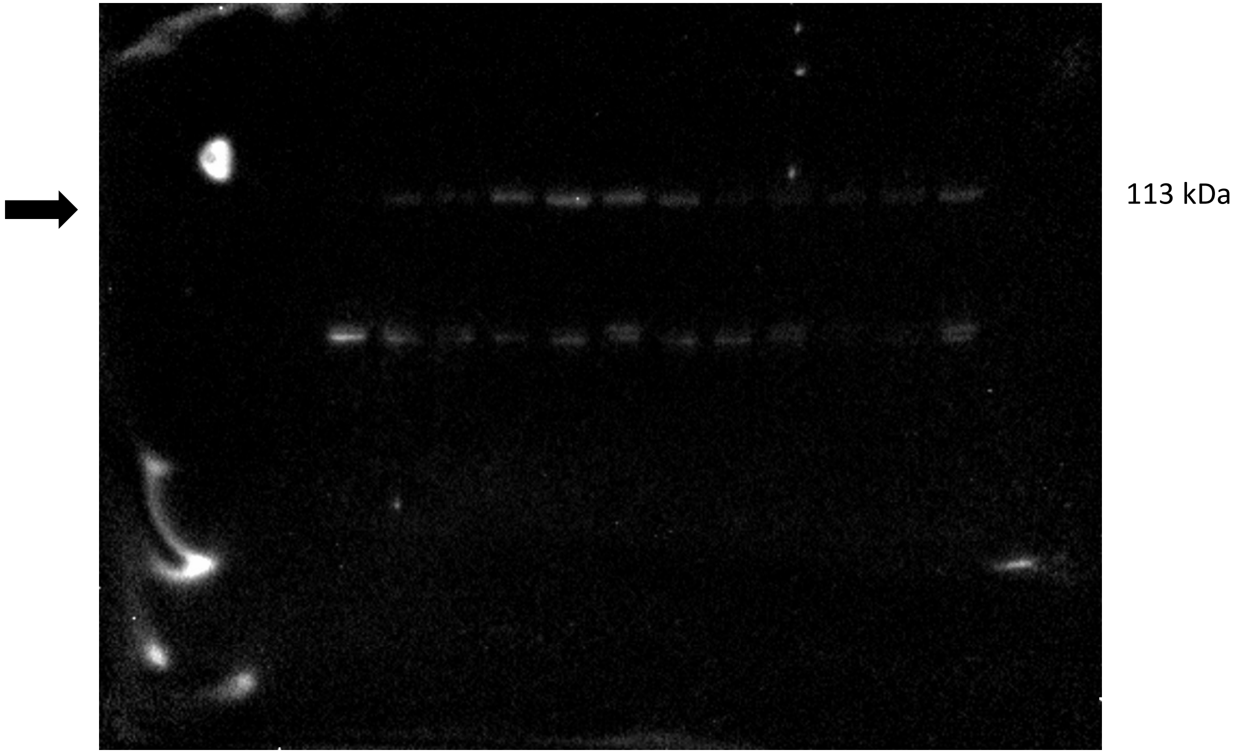

ASC

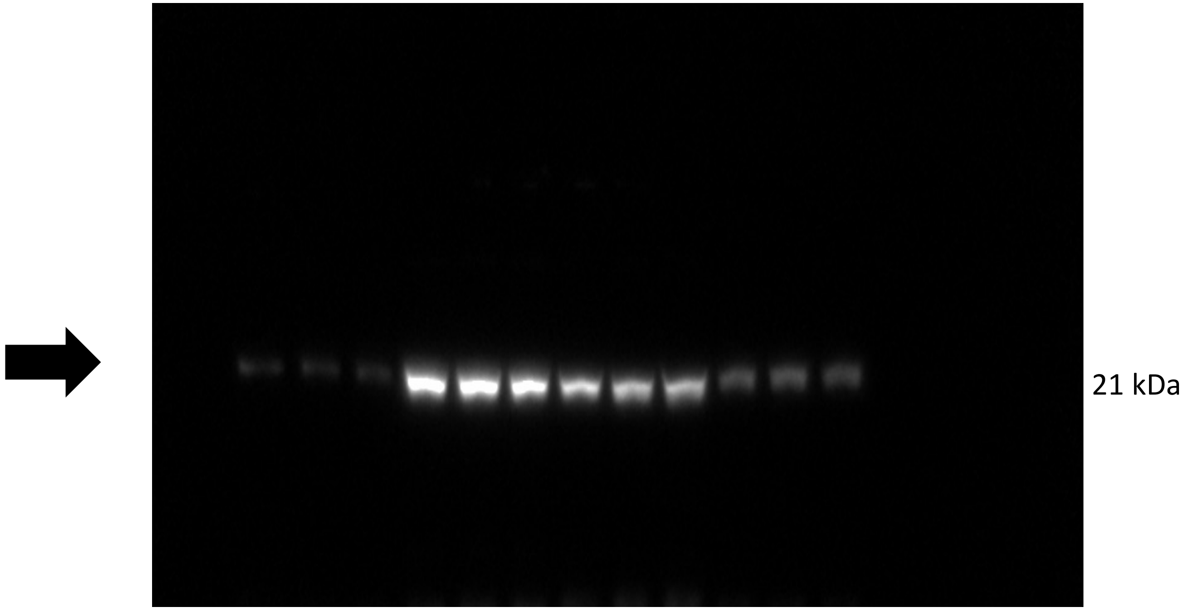

Original Western Blots
Lung samples

IL-18

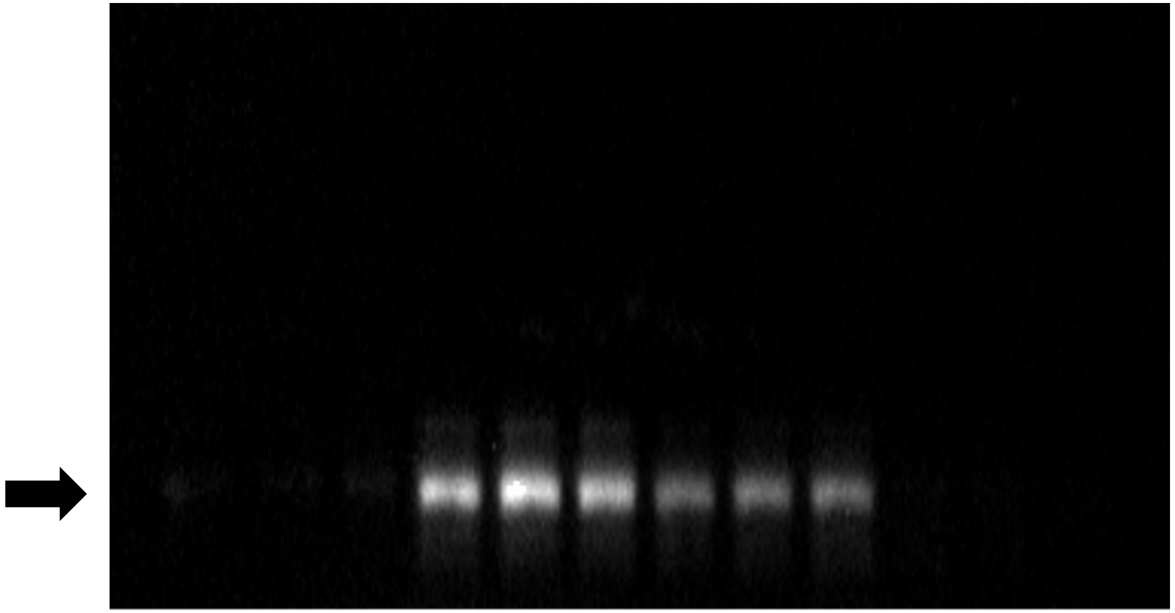

IL-1β

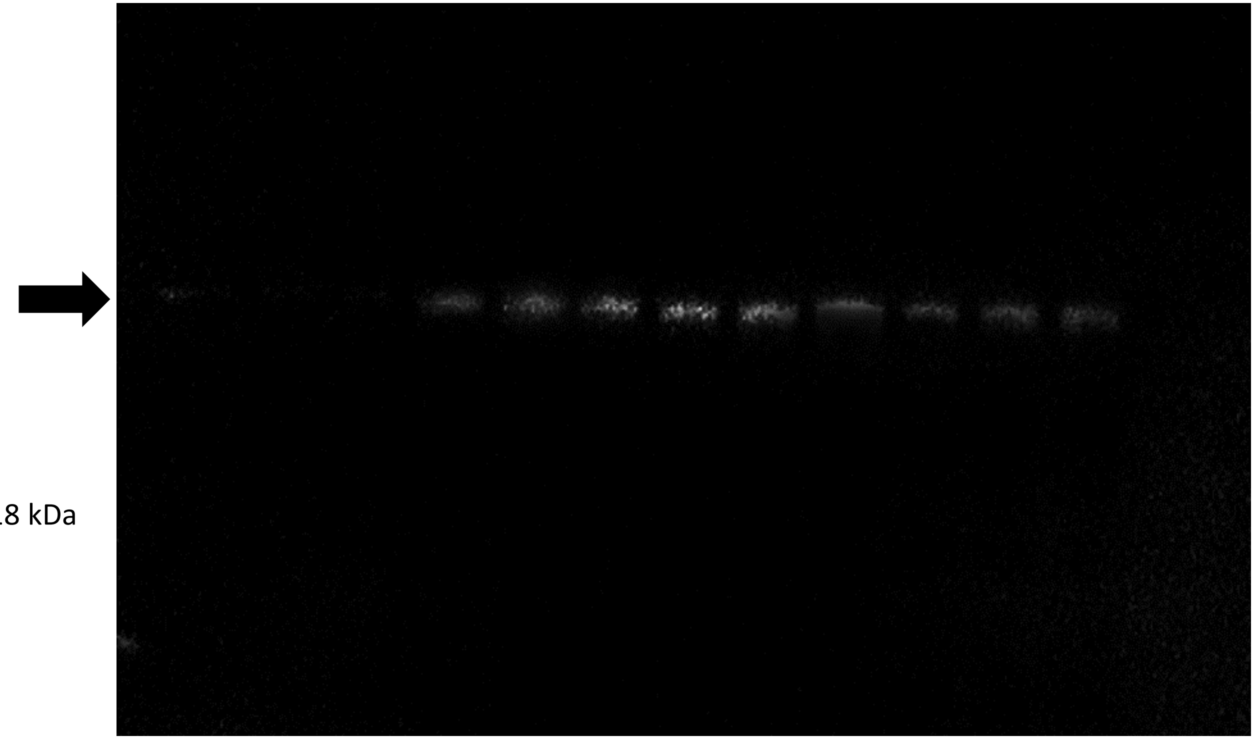

Original Western Blots
Lung samples

β -actin for NLRP3, ASC, IL-1β and IL-18

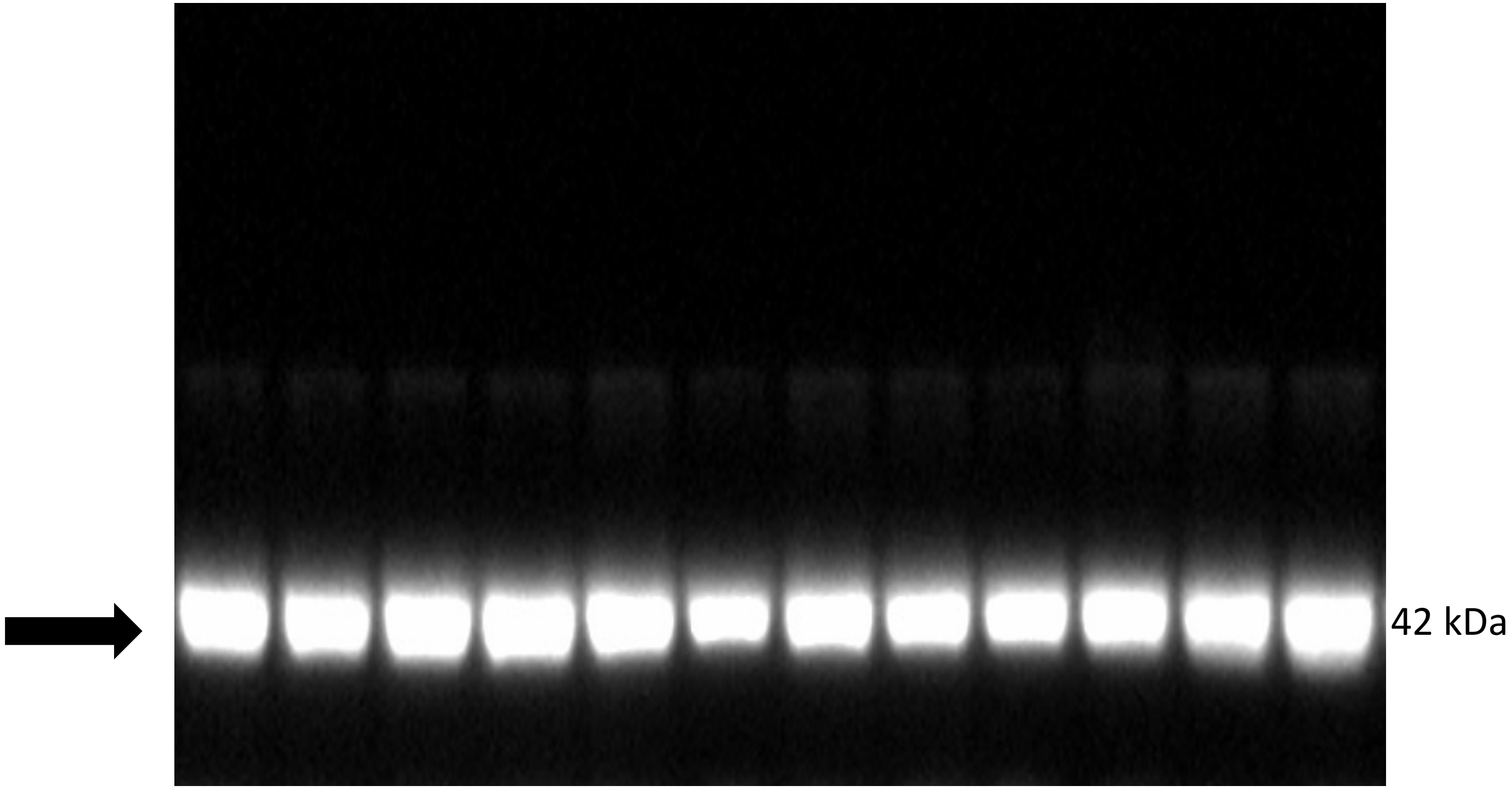

Original Western Blots
Spleen samples

NLRP3

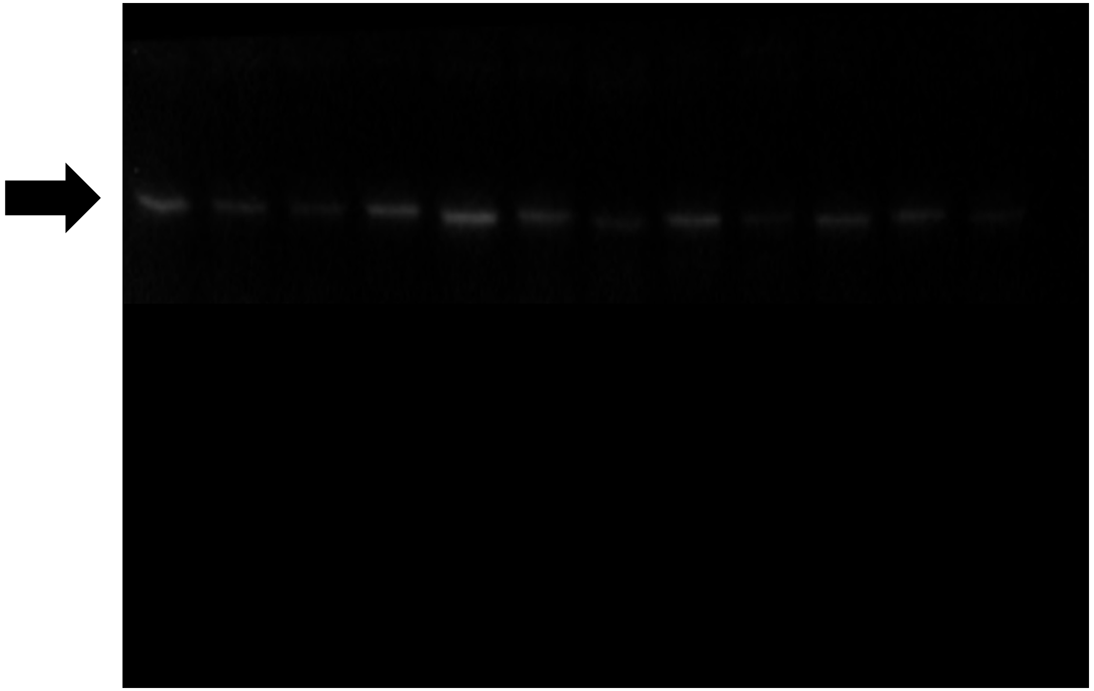

113 kDa

ASC

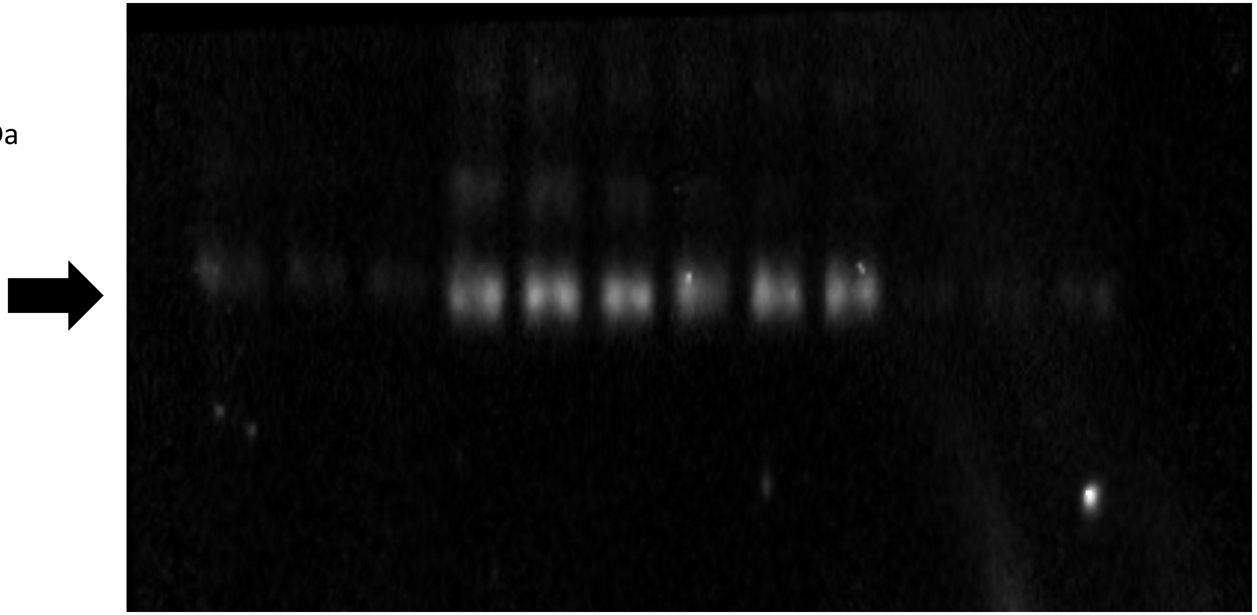

21 kDa

Original Western Blots
Spleen samples

IL-18

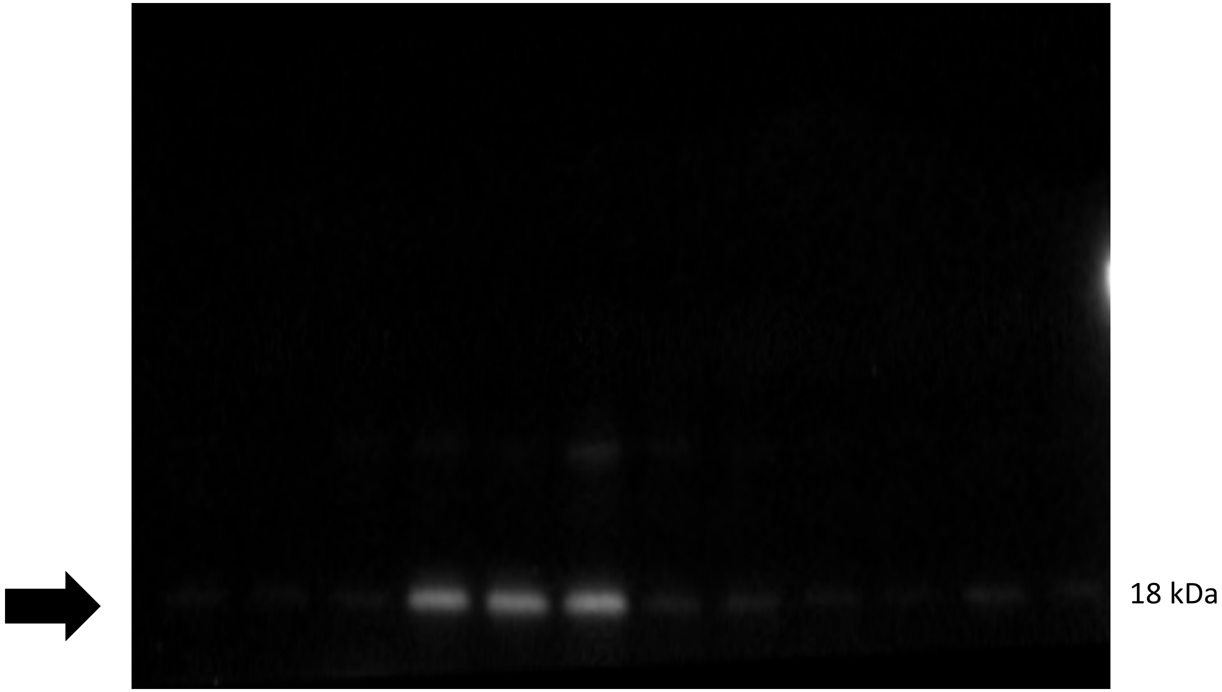

IL-1β

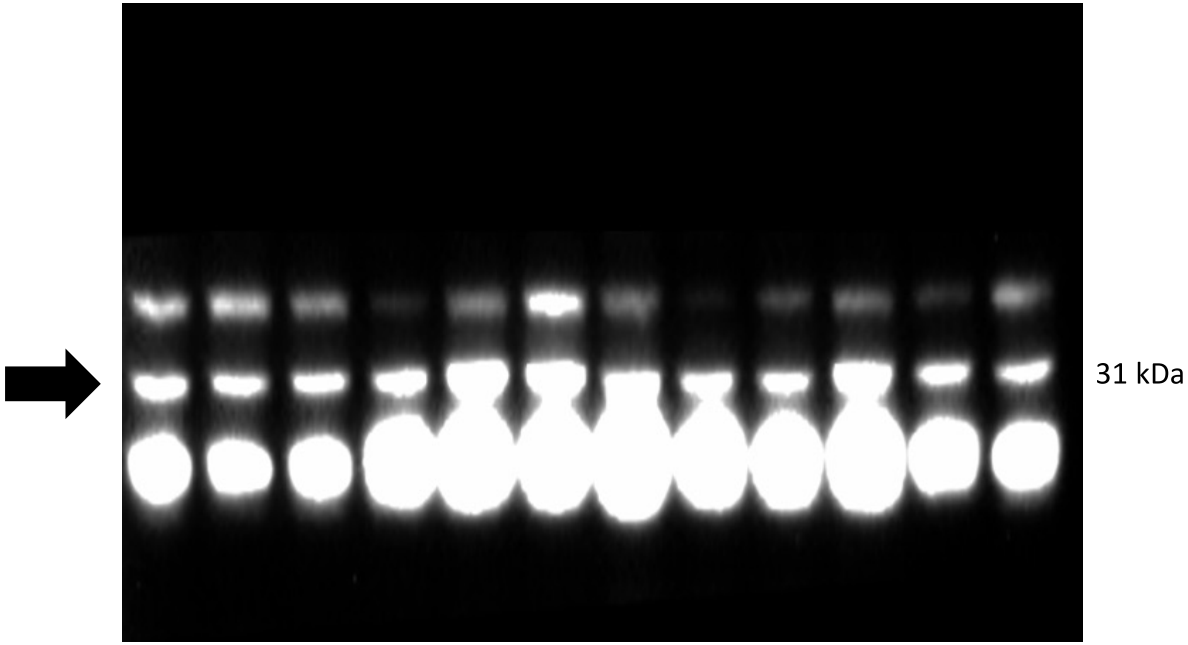

Original Western Blots
Spleen samples

β -actin for NLRP3, ASC, IL-1β and IL-18

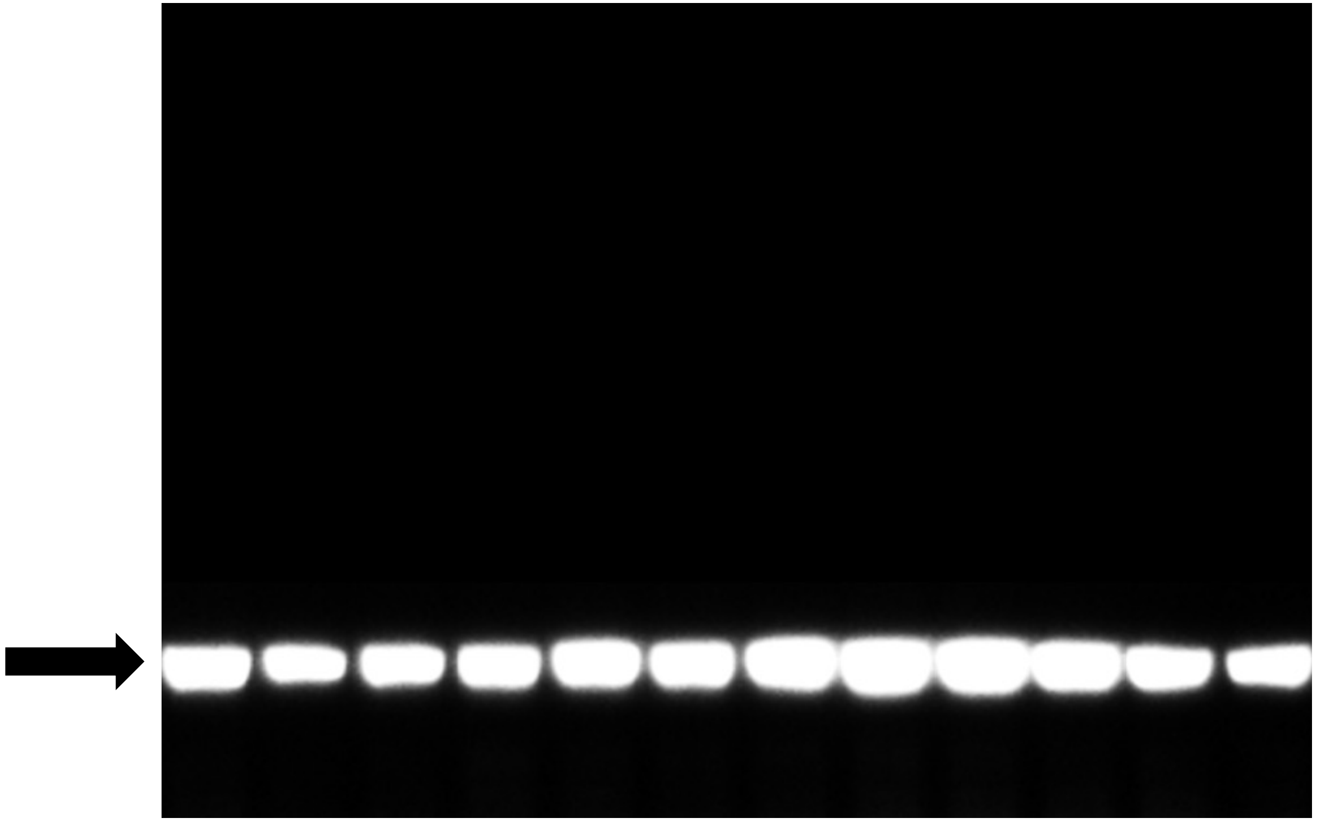

42 kDa
